# Study on the Separation of H_2_ from CO_2_ Using a ZIF-8 Membrane by Molecular Simulation and Maxwell-Stefan Model

**DOI:** 10.3390/molecules24234350

**Published:** 2019-11-28

**Authors:** Behrouz Bayati, Asma Ghorbani, Kamran Ghasemzadeh, Adolfo Iulianelli, Angelo Basile

**Affiliations:** 1 Department of Chemical Engineering, Ilam University, Ilam 69315-516, Iran; asmaghorbani94@gmail.com; 2 Faculty of Chemical Engineering, Urmia University of Technology, Urmia 7166-93187, Iran; kamran.ghasemzadeh@uut.ac.ir; 3 Institute on Membrane Technology of the Italian National Research Council (CNR-ITM), via P. Bucci cubo 17/C, 87036 Rende (CS), Italy; a.basile@itm.cnr.it

**Keywords:** ZIF-8 membrane, H_2_ separation, H_2_/CO_2_ permselectivity

## Abstract

The purification of H_2_-rich streams using membranes represents an important separation process, particularly important in the viewpoint of pre-combustion CO_2_ capture. In this study, the separation of H_2_ from a mixture containing H_2_ and CO_2_ using a zeolitic imidazolate framework (ZIF)-8 membrane is proposed from a theoretical point of view. For this purpose, the adsorption and diffusion coefficients of H_2_ and CO_2_ were considered by molecular simulation. The adsorption of these gases followed the Langmuir model, and the diffusion coefficient of H_2_ was much higher than that of CO_2_. Then, using the Maxwell–Stefan model, the H_2_ and CO_2_ permeances and H_2_/CO_2_ permselectivities in the H_2_–CO_2_ mixtures were evaluated. Despite the fact that adsorption of CO_2_ was higher than H_2_, owing to the simultaneous interference of adsorption and diffusion processes in the membrane, H_2_ permeation was more pronounced than CO_2_. The modeling results showed that, for a ZIF-8 membrane, the H_2_/CO_2_ permselectivity for the H_2_–CO_2_ binary mixture 80/20 ranges between 28 and 32 at ambient temperature.

## 1. Introduction

Energy has become one of the major concerns in the world due to the growing oil price and the concomitant depletion of fossil fuels, involving the need for greener processes and the use of renewable sources. Therefore, the search for renewable energy has attracted the attention of the scientific community [1,2]. In particular, H_2_ is seen as a pollution-free energy carrier, possessing high energy density and heat content 3 to 4 times higher than coal and natural gas. This simple element is currently used in chemical industries, particularly in methanol and ammonia production, food processing, and metallurgy, and for heating and electric power generation, in industrial boilers [3,4,5,6]. Unfortunately, H_2_ is not naturally available as a pure gas on the Earth and, at this time, approximately 90% of the total H_2_ production comes from non-renewable sources such as natural gas and coal, responsible for consistent greenhouse gas emissions [1,6]. Currently, H_2_ is produced predominantly by the natural gas steam reforming reaction in conventional reformers, followed by the water–gas shift (WGS) reaction, which provides a H_2_-rich stream containing also CO_2_ and other impurities [7]. Afterwards, these impurities must be removed in order to obtain high purity H_2_ for applications and downstream processes [6].

Pressure swing adsorption (PSA) as well as cryogenic and membrane separations have been considered effective methods to purify H_2_ from other gases such as CO_2_ [8,9]. In the framework of H_2_ purification by membrane technology and among the various types of membrane solutions, zeolite membranes possess special properties such as physical stability, appropriate chemical resistance, etc. [10]. Metal–organic frameworks (MOFs), as a new family of microporous membrane materials, are organized by a network of transition metal cations or clusters bonded by organic ligands [11]. Hence, MOFs have become interesting membrane materials, useful in applications such as gas separation, catalysis, and product storage, due to their feasibility in altering pore size and adsorption affinities by functionalizing the linked molecules as well as the porosity and functional groups [12,13]. Zeolitic imidazolate frameworks (ZIFs) are an important class of materials that can be categorized as MOFs. ZIF membranes include ZIF-7, ZIF-8, ZIF-22, ZIF- 90, ZIF-95, and ZIF-100, which show interesting performance in gas separation applications [14,15,16]. In particular, the separation performances of propylene/propane mixtures were investigated through a ZIF-8 membrane fabricated by a facile hydrothermal seeded growth method by Pan et al. [14]. They found that the ZIF-8 membrane had significant separation performance for a wide range of propylene/propane binary mixtures, showing high thermal and long-term stability, and high reproducibility. Lai et al. [15] studied the permeation of CO_2_/CH_4_ through a ZIF-8 membrane based on a combination of generalized Maxwell–Stefan, viscous flow, and Knudsen diffusion models, considering the gas diffusivity, support resistance, and intercrystalline pores of the membrane layer. They found that the simulated results and the experimental gas permeation data were well fitted and consistent with the physical characterizations, including scanning electron microscopy (SEM) and X-ray diffraction (XRD). Furthermore, the transport and diffusivities of hydrocarbons in ZIF-8 as a function of temperature were studied using molecular simulation methods via dynamically corrected transition state theory (dcTST) [16]. A comparison of the determined diffusivity results with experimental data demonstrated considerable agreement for all the molecules. Chokbunpiam et al. [17] evaluated the adsorption, diffusion, and permeation of the guest molecules in the C_2_H_6_/ZIF-8 system and the influence of the diffusing C_2_H_6_ molecules in the ZIF-8 membrane by molecular dynamic simulation. They found that two effects simultaneously include the decrease in window size of the ZIF-8 membrane at higher C_2_H_6_ loadings, while exerting forces within the cavity by the guest molecules at higher loadings, pressing a given probe molecule toward the window and leading to a weak self-diffusivity dependence on the concentration of guest molecules.

From the perspective of pre-combustion capture and related zeolite membrane application, the present theoretical study aimed to study the adsorption and diffusion coefficients of H_2_ and CO_2_ in a ZIF-8 membrane, calculated by using molecular simulations. Then, using the results of molecular simulations and the Maxwell–Stefan model, the membrane permeance of H_2_ and CO_2_ binary mixtures was also investigated and is discussed below.

## 2. Materials and Methods

### 2.1. Molecular Simulation Details

**Figure 1:**
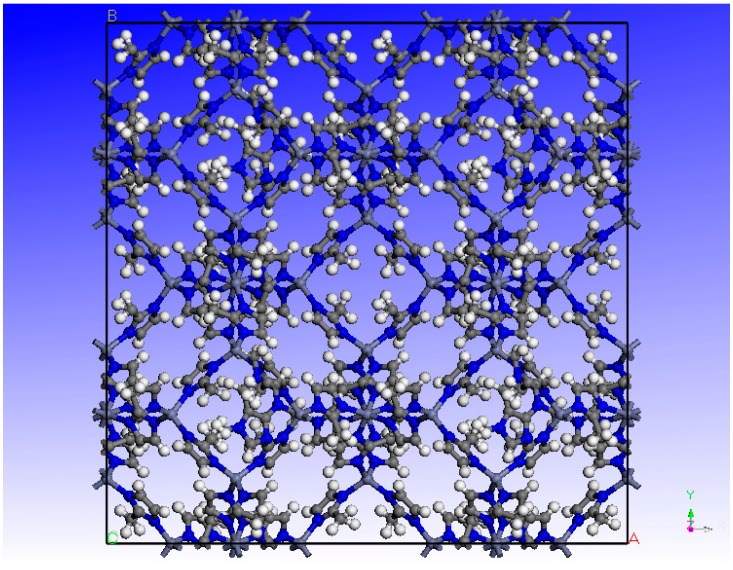
Schematic diagram of the supercell of the ZIF-8 structure.

The Materials Studio software (BIOVIA, San Diego, CA, USA) was used for the molecular simulation of adsorption and diffusion of H_2_ and CO_2_ in a ZIF-8-based membrane. The universal force field was used in all the simulations. The structure of the simulated ZIF-8-based membrane of this study was assumed to consist of a supercell with dimensions of 34 × 34 × 34 Å, as shown in Figure 1. The sorption modulus was used to simulate the adsorption of the components and the Monte Carlo method with periodic boundary conditions was applied to simulate the adsorption on the ZIF-8-based membrane. The cutoff distance in the calculations was 12.5 Å, electrostatic and van der Waals terms were Ewald and atom-based, respectively. The number of calculations was up to a balance of 1,000,000 stages and production steps of about 106. Considering that the driving force for the movement of adsorbed molecules between phases is expressed by their fugacity, the Molecular Dynamics simulation was used to investigate the diffusion of the components in the structure of the ZIF-8-based membrane. First, 10 molecules of a component were located in a ZIF-8 membrane cell by the sorption module. Then, the system was minimized using the conjugate gradient and steepest descent methods. Molecular Dynamics was carried out 1000 ps NVT at 298 K to reach the equilibrium state. The diffusion coefficients were derived from the linear least-square fits of the plots of the mean square displacement (MSD) of molecules versus time.

### 2.2. Maxwell–Stefan Model

The transport through the ZIF-8-based membranes was demonstrated as adsorption on the external surface, transport into the pores, intercrystalline diffusion, transport out of the pores, and desorption. Different mechanisms may contribute to the selectivity of the ZIF-8 membranes; indeed, in their pores, adsorption equilibrium and diffusion play a major role for some molecules and in particular conditions, whereas for others the molecular sieve effects turn out to be dominant. Considerable progress has been made during the last decade in developing a general theory for describing the diffusion of gaseous mixtures in zeolite membranes, using the Maxwell–Stefan (M–S) formulation [18,19], which is considered an indispensable model for simulating the transient transport across zeolites membranes, to be strongly preferred to other modeling approaches (for example, the simple Fick model) [20,21,22,23,24,25,26]. Therefore, it is now widely accepted that, for a proper formulation, it can be applied for describing the gas diffusion in zeolite membranes. It is also generally accepted that the fundamentally correct approach is related to the fluxes (Ni), defined in terms of the cross-sectional area of the membrane, and to the chemical potential gradients (∇µi), by using the M–S equation, shown as Equation (1) below [27]:

(1) $$-\rho \frac{\Theta_{i}}{RT}\nabla \mu_{i}=\sum_{\begin{array}{c} j=1\\ j\neq i \end{array}}^{n}\frac{\Theta_{j}N_{i}-\Theta_{i}N_{j}}{\Theta_{i,sat}\Theta_{j,sat}{Đ}_{ij}}+\frac{N_{i}}{\Theta_{i,sat}{Đ}_{i}}.$$

The friction is the result of the interactions between adsorbed molecules and between a molecule and the pore wall. The parameters Đ_ij_ and Đ_i_ are the Maxwell–Stefan surface diffusivities and represent inverse friction factors between molecules and between molecules and pore wall, respectively, whereas Θ_i_ is the molecule loading expressed in molecules per unit cell and Θ_i,sat_ is the saturation loading. The interchange coefficient Đ_ij_ is calculated as a logarithmic average of the single-component M-S diffusivities, Equation (2):

(2) $${Đ}_{ij}={\left[{Đ}_{i}\right]}^{\frac{\theta_{i}}{\theta_{i}+\theta_{j}}}{\left[{Đ}_{j}\right]}^{\frac{\theta_{j}}{\theta_{i}+\theta_{j}}}.$$

The fractional occupancies are defined by Equation (3):

(3) $$\theta =\frac{q_{i}}{q_{i}^{sat}}=\frac{\Theta_{i}}{\Theta_{i}^{sat}},$$

where *q_i_* is the molar loading of species i and *q_i,_^sat^* is its saturation loading.

The chemical potential gradient may be expressed in terms of the fractional occupancy gradient via thermodynamic correction factors Γ_ij_, defined by Equations (4) and (5):

(4) $$\frac{\theta_{i}}{RT}\nabla \mu_{i}=\sum_{j=1}^{n}\Gamma_{ij}\nabla \theta_{j},$$

(5) $$\Gamma_{ij}=\theta_{i}\frac{\partial lnp_{i}}{\partial \theta_{j}}.$$

The adsorption of components in equilibrium with the ZIF-8-based membrane can be described by a Langmuir adsorption isotherm equation (Equation (6):

(6) $$\Theta =\frac{b\Theta_{sat}P\;\;}{1+bP},$$

where *P* is the pressure (Pa) and *b* represents the temperature-dependent sorption strengths (expressed in Pa^−1^) [25].

## 3. Results and Discussion

**Figure 2:**
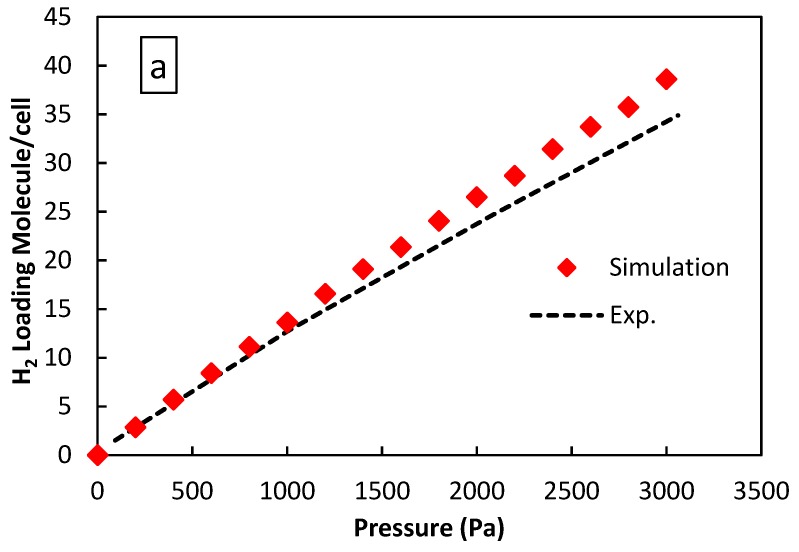

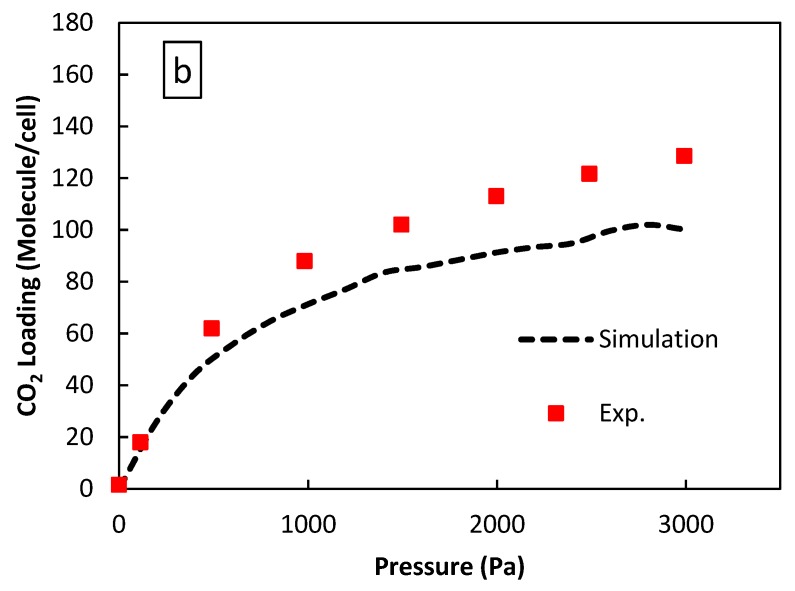
Comparisons between the simulated isotherm adsorption and experimental results from the literature on the ZIF-8-based membrane at 298 K: (**a**) H_2_ (experimental results from [26]) and (**b**) CO_2_ (experimental results from [27]).

Figure 2 shows the simulated adsorption isotherms for H_2_ and CO_2_ on zeolite ZIF-8 at 298 K and their comparisons with the experimental isotherms from the literature [28,29]. The agreement between the simulated isotherms and the experimental results coming from the literature of H_2_ and CO_2_ adsorption in the entire range of pressure studied in this work is reported in Figure 2a,b. On the one hand, the molecular simulations of H_2_ adsorption match quite well the experimental results apart from the range of pressures higher than 2500 Pa where a certain disagreement is evident, as is seen in Figure 2a. On the other hand, the molecular simulations of CO_2_ adsorption match quite well the experimental data from the literature at lower pressures, whereas at higher pressures a clear deviation from the simulated isotherms is evident toward lower loadings, as shown in Figure 2b. This occurs because, at higher pressures, the system is far from the ideal state and the non-ideal terms are not incorporated into the system, with a consequent disagreement. Furthermore, at high pressures, some structural features such as the simulated surface area, pore volume of the adsorbent and force field parameters can have an influence on the agreement within experimental and modeling data. However, both figures indicate the validity of the simulation results using the universal force field.

**Figure 3:**
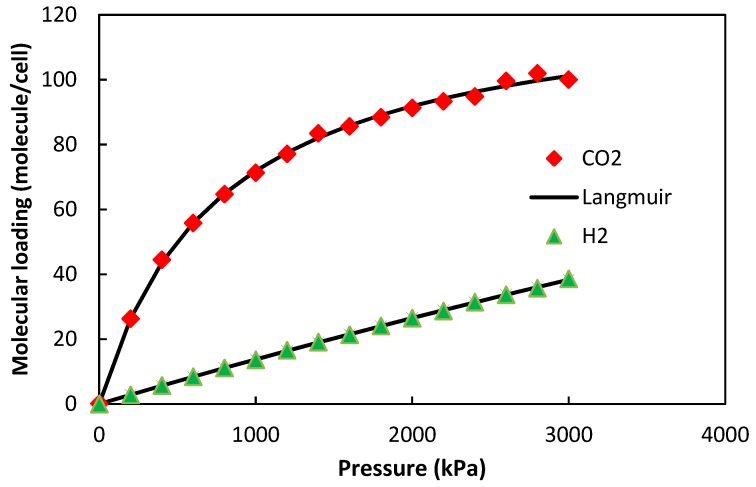
Langmuir model fitting to the molecular simulation results of H_2_ and CO_2_ uptake on the ZIF-8-based membrane at 298 K.

In Figure 3, simulation results of H_2_ and CO_2_ adsorption on the ZIF-8-based membrane were fitted by using a Langmuir model. As shown, there is a good agreement between the former model and the molecular simulation results. In particular, the adsorption of H_2_ and CO_2_ on the ZIF-8-based membrane looks like a monolayer adsorption. The model parameters derived from the fitting process are shown in Table 1.

**Figure 4:**
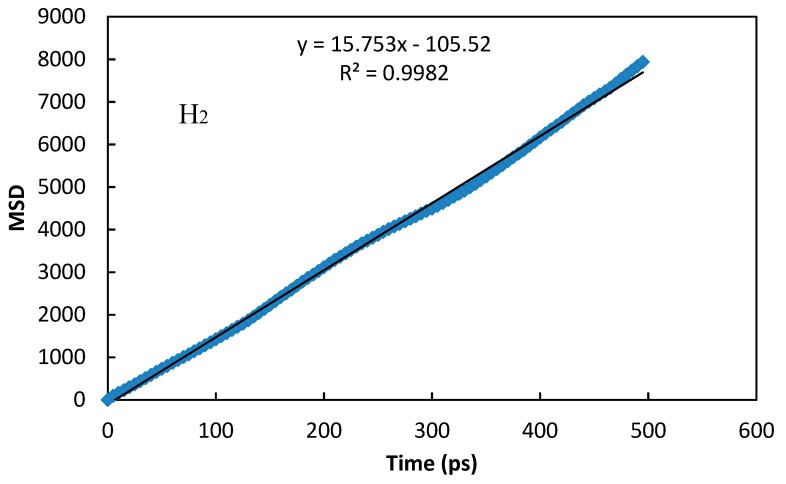

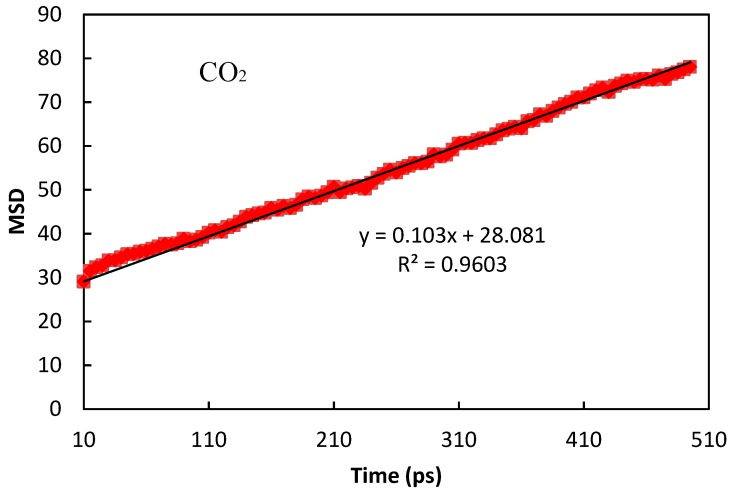
Mean square displacement (MSD) as a function of time for both H_2_ and CO_2_ molecule diffusion in the ZIF-8-based membrane at 298 K and for a set loading of 10 molecules/cell.

MSDs were evaluated from trajectories that are carried out through the periodic boundaries [30]. Figure 4 displays the MSD as a function of time for both H_2_ and CO_2_ molecule diffusion in the ZIF-8-based membrane at 298 K and for a set loading of 10 molecules/cell. The relationship between the observed MSD versus time is linear, with a very good approximation, as also observed in other works in the literature [31,32].

This indicates that the normal diffusion occurs at this time scale. The calculated diffusion coefficient at 298 K for a set loading of 10 molecules is equal to 2.62 × 10^−8^ for H_2_ and 1.71 × 10^−10^ m^2^⋅s^−1^ for CO_2_, respectively. In both cases, it is calculated as the slope of the plot MSD versus time, as is shown in Figure 4. These values are in good agreement with reported experimental diffusion coefficients in the literature [28,29] (Table 1). Table 2 shows further H_2_ and CO_2_ diffusion coefficients at 298 K of other membranes beside the ZIF-8 one of this study.

As shown, in most of the cases reported in the table, H_2_ and CO_2_ diffusion coefficients of the ZIF-8 membrane are higher than the other ones from the literature, apart from the H_2_ diffusion from [34] and the CO_2_ diffusion from [33].

The molecular simulation results of the adsorption and diffusion of H_2_ and CO_2_ in the ZIF-8-based membrane showed that the latter adsorption was much higher than that of H_2_. On the other hand, the H_2_ diffusion coefficient in the ZIF-8-based membrane was much higher than that of CO_2_. Since the permeance of these components across the ZIF-8 membrane depends on their adsorption and diffusion properties, the Maxwell–Stefan model was adopted for investigating these phenomena.

**Figure 5:**
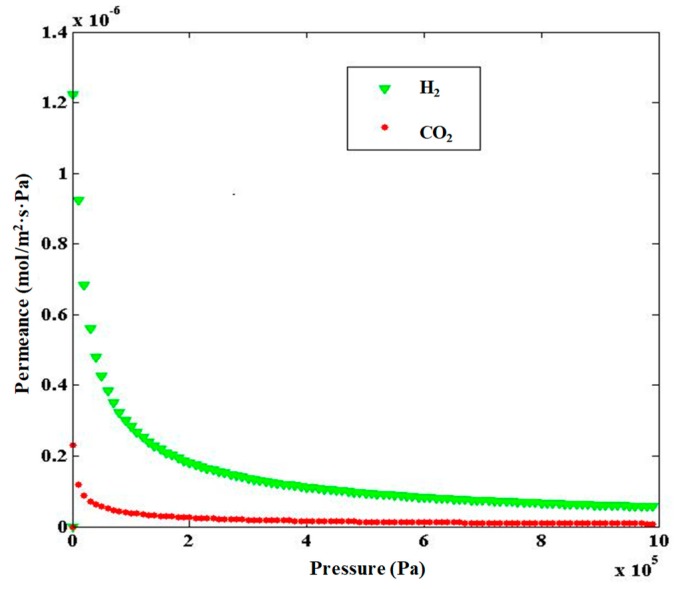
Permeance of H_2_ and CO_2_ in a binary mixture H_2_/CO_2_ = 70/30 for the ZIF-8-based membrane at 298 K and as a function of pressure.

**Figure 6:**
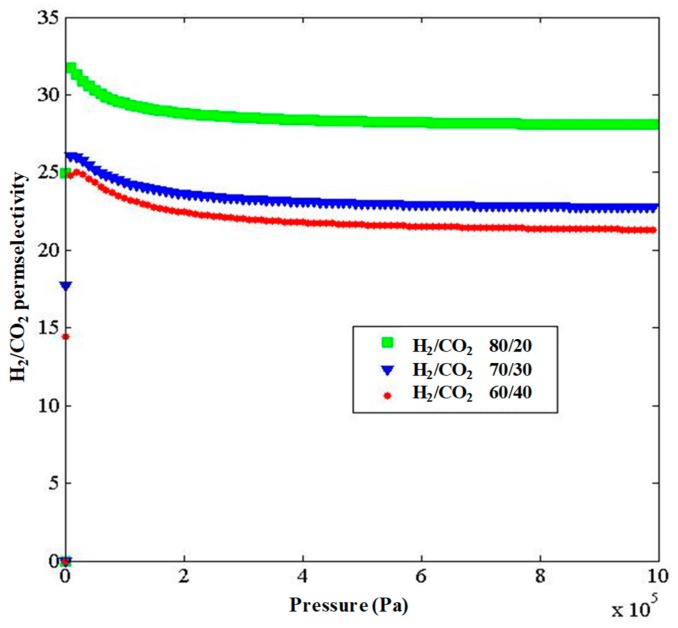
H_2_/CO_2_ selectivity of the ZIF-8 membrane for the binary H_2_–CO_2_ mixture at 298 K and different H_2_/CO_2_ molar ratios.

In Figure 5, the permeances of H_2_ and CO_2_ as a binary mixture (H_2_/CO_2_ molar ratio = 70/30) are shown at a temperature of 298K and different pressures. It is observable that H_2_ permeance is higher than CO_2_, particularly at lower pressures, making the ZIF-8-based membrane suitable for H_2_ separation from CO_2_. H_2_/CO_2_ membrane selectivities for various H_2_–CO_2_ mixtures at different molar ratio were simulated as a function of pressure at 298 K, as seen in Figure 6. It can be observed that by increasing the H_2_ concentration, the H_2_/CO_2_ selectivity increased as a consequence of a higher hydrogen permeation driving force across the membrane. Meanwhile, the CO_2_ molecule density was reduced, causing their adsorption decrease in the competition with other molecules. Furthermore, at lower pressures, H_2_/CO_2_ permselectivity was higher because CO_2_ showed lower adsorption at low pressures and less effect on permeance; this mode is more favorable for hydrogen selection. As the best result of this theoretical work, Figure 6 shows that the highest simulated H_2_/CO_2_ permselectivity was reached with the H_2_–CO_2_ mixture showing a H_2_/CO_2_ ratio equal to 80/20, with values ranging between 28 and 32, although at a pressure higher than 300 kPa the permselectivity value showed a constant trend around 28.

## 4. Conclusions

The separation of H_2_ from CO_2_ was theoretically investigated by using a ZIF-8 membrane. The adsorption and diffusion contributions of H_2_ and CO_2_ were studied by using molecular simulations. The ZIF-8-based membrane showed a strong tendency to adsorb CO_2_, whereas the H_2_ diffusion coefficient was much higher than that of CO_2_. By combining the molecular simulation results with the Maxwell–Stefan model, the theoretical results demonstrated that the ZIF-8 membrane possesses a H_2_/CO_2_ permselectivity higher than 30 at relatively lower pressure (below 300 kPa), while it decreases raising the pressure. This effect is due to the progressively reduced CO_2_ adsorption contribution at lower pressure. However, this trend was theoretically confirmed in all the H_2_–CO_2_ binary mixtures considered in this work.

## Figures and Tables

**Table 1:** Obtained Langmuir isotherm parameters and diffusion coefficient of H_2_ and CO_2_ on the ZIF-8-based membrane at 298 K.

Component	Langmuir Model	R^2^	Diffusion Coefficient (m^2^/s)	
			This Work	Literature
H_2_	Θs = 356; b = 4.02 × 10^−5^	0.999	2.62 × 10^−8^	2.5 × 10^−8^ [28]
CO_2_	Θs = 127; b = 1.31 × 10^−3^	0.998	1.71 × 10^−10^	2.2 × 10^−10^ [29]

**Table 2:** H_2_ and CO_2_ diffusion coefficients at 298 K for the ZIF-8 membrane of this study and those of other membranes from the literature.

D_H2_ [m^2^/s]	D_CO2_ [m^2^/s]	Membrane	Ref.
2.62 × 10^−8^	1.71 × 10^−10^	ZIF-8	This work
1.73 × 10^−8^	5.22 × 10^−10^	Silicalite	[33]
5.06 × 10^−8^	1.45 × 10^−10^	Silicalite	[34]
1.33 × 10^−9^	8.82 × 10^−11^	DDR zeolite	[35]
5.01 × 10^−9^	1.58 × 10^−11^	DDR zeolite	[36]
5.79 × 10^−10^	4.12 × 10^−11^	NaY zeolite	[37]
1.27 × 10^−9^	1.23 × 10^−10^	SAPO-34	[38]
